# Fitness correlates of blubber oxidative stress and cellular defences in grey seals (*Halichoerus grypus*): support for the life-history-oxidative stress theory from an animal model of simultaneous lactation and fasting

**DOI:** 10.1007/s12192-023-01332-1

**Published:** 2023-03-18

**Authors:** Holly C. Armstrong, Debbie J. F. Russell, Simon E. W. Moss, Paddy Pomeroy, Kimberley A. Bennett

**Affiliations:** 1 Marine Biology and Ecology Research Centre, Plymouth University, Drake Circus, Plymouth, PL4 8AA UK; 2 School of Psychology and Neuroscience, University of St Andrews, St Andrews, KY16 9JP UK; 3 Sea Mammal Research Unit, Scottish Oceans Institute, University of St Andrews, St Andrews, KY16 8LB UK; 4 Division of Health Science, School of Applied Sciences, Abertay University, Dundee, DD1 1HG UK

**Keywords:** Cellular stress, Cellular chaperone, Adipose, Trade off, Pinniped

## Abstract

Life-history-oxidative stress theory predicts that elevated energy costs during reproduction reduce allocation to defences and increase cellular stress, with fitness consequences, particularly when resources are limited. As capital breeders, grey seals are a natural system in which to test this theory. We investigated oxidative damage (malondialdehyde (MDA) concentration) and cellular defences (relative mRNA abundance of heat shock proteins (*Hsps*) and redox enzymes (*REs*)) in blubber of wild female grey seals during the lactation fast (*n* = 17) and summer foraging (*n* = 13). Transcript abundance of *Hsc70* increased, and *Nox4*, a pro-oxidant enzyme, decreased throughout lactation. Foraging females had higher mRNA abundance of some *Hsps* and lower *RE* transcript abundance and MDA concentrations, suggesting they experienced lower oxidative stress than lactating mothers, which diverted resources into pup rearing at the expense of blubber tissue damage. Lactation duration and maternal mass loss rate were both positively related to pup weaning mass. Pups whose mothers had higher blubber glutathione-S-transferase (*GST*) expression at early lactation gained mass more slowly. Higher glutathione peroxidase (*GPx*) and lower catalase (*CAT*) were associated with longer lactation but reduced maternal transfer efficiency and lower pup weaning mass. Cellular stress, and the ability to mount effective cellular defences, could proscribe lactation strategy in grey seal mothers and thus affect pup survival probability. These data support the life-history-oxidative stress hypothesis in a capital breeding mammal and suggest lactation is a period of heightened vulnerability to environmental factors that exacerbate cellular stress. Fitness consequences of stress may thus be accentuated during periods of rapid environmental change.

**Supplementary Information:**

The online version contains supplementary material available at 10.1007/s12192-023-01332-1.

## Introduction

The ability to mount an adequate stress response dictates whether an organism survives and thrives during physiological challenges (Parsons 2005; Blas et al. 2007). Stress responses divert resources from essential functions to deal with immediate requirements, creating a trade-off in allocation to energy-consuming processes when resources are limited (Alonso-Alvarez et al. 2004; Wiersma et al. 2004; Parsons 2005; Blas et al. 2007). Stress is therefore a powerful selective force, acting at cell and organism levels to influence life histories and population dynamics (von Schantz et al. 1999; Hoffmann and Hercus 2000; Alonso-Alvarez et al. 2004; Monaghan and Spencer 2014).

It has been postulated that life history trade-offs are mediated in part by oxidative stress (Costantini 2008; Garrett et al. 2011; Speakman et al. 2015), induced by reactive species (RS) generated by aerobic respiration and pro-oxidant enzyme activity (Lambeth 2004). RS are important cell signals (Hancock et al. 2001; Sies 2017), but cause damage when present in excess (Valko et al. 2007). Cellular stress can also cause proteins to unfold, become non-functional and/or form cytotoxic aggregates (Fulda et al. 2010). Intracellular redox enzymes (RE) and heat shock proteins (HSPs) minimise and mitigate against oxidative and proteotoxic stress through their antioxidant and molecular chaperone functions (Feder and Hofmann 1999; Pamplona and Constantini 2011). Induction of cellular defences can increase resilience to current and subsequent stressors (Feder et al. 1996; Feder and Hofmann 1999). However, costs of their synthesis and activity (Hoekstra and Montooth 2013) are harder to meet when resources are limited.

Life-history-oxidative stress theory predicts that allocation of resources to cellular maintenance, protection and repair, such as RE and HSPs, is lowered during life history stages that require increased energy demand, particularly during resource limitation, resulting in greater oxidative damage and negative effects on future survival and reproductive output (Costantini 2008; Garrett et al. 2011; Speakman et al. 2015; Al Jothery et al. 2016). The impact of external environmental sources of stress, such as rapid climate change and pollution, may thus impact most heavily on life history stages already facing a trade-off in resource allocation to cellular defences and other essential processes.

Lactation increases energetic requirements by twofold to sixfold (Prentice and Prentice 1988; Crocker et al. 2001). Although females of some species have greater cellular defences to protect against oxidative stress during reproduction (Viña et al. 2006; Borras et al. 2007), including lactation (Pichaud et al. 2013), buffering against oxidative damage is not always possible in all tissues (Bergeron et al. 2011; Zhao et al. 2015; Stier et al. 2017; Perrault and Stacy 2018) and elevated costs cannot always be matched by increased food intake, resulting in resource limitation and trade-offs in investment in defences. Starvation or fasting itself can be a pro-oxidant state (Sorensen et al. 2006; Salin et al. 2018), even in fast-adapted species (Sharick et al. 2015; Schull et al. 2016; Colominas-Ciuró et al. 2017), and can increase HSP levels or expression (Ehrenfried et al. 1996; Heydari et al. 1996). Fasting during lactation may thus be particularly challenging due to increased oxidant production when resources are limited. Conversely, fasting can increase cellular protection and reduce oxidative stress (Longo and Mattson 2014; Ensminger et al. 2021). Fast-adapted species may also buffer oxidant production very effectively (Colominas-Ciuró et al. 2017; Stier et al. 2019; Ensminger et al. 2021). For example, elephant seal pups increase cellular defences and thus avoid oxidative stress in some tissues during fasting (Vázquez-Medina et al. 2010, 2011; Martinez et al. 2018). Similarly, HSP70 and glutathione levels are higher in liver and blubber of suckling grey seal (*Halichoerus grypus*) pups compared to when they are fasting (Bennett et al. 2014), suggesting either greater physiological stress occurs during rapid mass gain or that suckling pups have greater resource to invest in cellular defence.

Capital breeders may avoid or mitigate impacts of resource limitation because they accumulate energy as large fat stores prior to breeding (Houston et al. 2006). Indeed, greater energy stores are associated with lower oxidative damage in long distance migrating birds (Eikenaar et al. 2020a). High rates of food intake and rapid fat deposition during refuelling are associated with increased antioxidant capacity, which limits oxidative damage (Skrip et al. 2015; Eikenaar et al. 2016, 2020b). However, rapid deposition of large fat stores can cause adipose tissue inflammation and reduction in HSP70 in humans and rodents (Furukawa et al. 2004; Grimsrud et al. 2007; Di Naso et al. 2015; Masschelin et al. 2020). It is unclear whether fasting during lactation is associated with greater cellular stress and reduced capacity for defence compared with periods of rapid weight gain in capital breeding mammals. Fasting, lactating females may be particularly vulnerable to rapid environmental changes that increase requirements for cellular defence, such as increased temperatures on the breeding colony, reduced food availability in fattening phases and/ or pollutant exposure that causes oxidative or proteotoxic stress.

Many studies focus on antioxidant defences in plasma, immune cells and active tissues such as the liver, brain and brown adipose (Eikenaar et al. 2016, 2020a, b; Zhao et al. 2015). There is less information on trade-offs in oxidative defences in white adipose tissue (WAT), likely because it has a low rate of oxygen consumption compared to other tissues (Goossens and Blaak 2012; Lempesis et al. 2020). However, WAT is vital in energy balance regulation, and the appropriate accumulation of fat stores and their normal function is a key determinant of fitness in many animals (Atkinson and Ramsay 1995; Pomeroy et al. 1999; Hall et al. 2001; Vervaecke et al. 2005; Miller et al. 2011). WAT is a target of oxidative stress and inflammation in humans during mass gain (Furukawa et al. 2004; Grimsrud et al. 2007; Di Naso et al. 2015; Masschelin et al. 2020), and lipids are highly susceptible to peroxidation by RS (Rikans and Hornbrook 1997). RS are, however, important regulators of lipolysis in WAT (Krawczyk et al. 2012; Abou-Rjeileh and Contreras 2021), such that a fine balance is needed between their production and elimination to maintain fatty acid supply during fasting. Blubber, the specialised subcutaneous WAT in marine mammals, is essential for insulation, metabolic fuel for maintenance and fat for milk synthesis during lactation. Cellular defences and oxidative stress in adipose tissue have not been investigated widely in mammals that undergo large and rapid changes in fat depot size during natural feeding and fasting, but may contribute to changes in their health and resilience during annual cycles, and constrain life history decisions that impact on current or future fitness that rely on healthy fat tissue, such as allocation of resources to rearing of young.

Grey seals are an ideal natural system in which to test the life-history-oxidative stress theory because they are typical mammalian capital breeders. Female grey seals increase in fat content from 12% after moulting to ~ 33% prior to parturition (Sparling et al. 2006; Hanson et al. 2019). Grey seal mothers fast during their ~ 18–21-day lactation, when they rely on blubber lipid for metabolic and milk production requirements and lose 40% of initial mass and 61–84% of fat reserves (Fedak and Anderson 1982; Pomeroy et al. 1999). We investigated whether grey seal mothers reduce their blubber cellular defences during lactation fasting and have lower levels in comparison to (presumed) pregnant foraging females. We also explored the associations between cellular defences and markers of oxidative damage during lactation with maternal performance. Specifically, we investigated (1) blubber relative *Hsp* and *RE* mRNA abundance, and malondialdehyde (MDA) levels during lactation to explore the association between allocation to defences and/ or lipid peroxidation and diminishing fat reserves; (2) whether larger animals have more robust defences; (3) differences in blubber HSP and RE mRNA abundance, and MDA levels between presumed pregnant, foraging females and mothers during the lactation-fast to determine which life history stage experiences greater oxidative stress; and (4) whether increased blubber cellular stress or damage is associated with reduced maternal performance metrics.

## Methods

Wild adult female grey seals were studied on the Isle of May, Scotland (56° 11′ 25″ N, 02° 34′ 25″ W) from October to December 2013 (fasting mothers; *n* = 18) and at Blakeney, (52° 57′ 58.32″ N, 0° 57′ 46.70″ E) and Donna Nook, east England (53° 28′ 33.12″ N, 0° 08′ 27.02″ E) in early May 2015 (foraging (presumed pregnant) females; *n* = 13). Capture and handling procedures were performed by personal licence holders or designated competent personnel under United Kingdom (UK) Home Office project licence #60/4009 and conformed to the UK Animals (Scientific Procedures) Act, 1986 and the European Union (EU) Directive 2010/63/EU on the protection of animals used for scientific procedures. The research was approved by the University of St. Andrews Animal Welfare and Ethics Committee.

### Animal capture and handling

#### Fasting females and maternal performance

Mothers, identified by a brand or flipper tag (Dalton ID Systems, Henley on Thames, Oxon, UK), were observed daily on the colony to record birth and weaning dates of their pups. Mother–pup pairs were captured twice: at ~ day 6 (6.5 ± 2; early) and at ~ day 17 (17.5 ± 0.5; late) of suckling. At each capture, mothers were anaesthetised with an estimated mass specific dose of 1 mL 100 kg ^−1^ intramuscular Zoletil^100^ (Virbac, Carros, France) (Pomeroy et al. 1999), delivered via blowpipe using a pressurised projectile syringe, aimed at the posterior lumbar muscles (Baker et al. 1990; Langton et al. 2011). At first capture, pup sex was recorded and a tag attached to the interdigital webbing of each rear flipper (Fedak and Anderson 1982). Mass, nose-tail length and standard axial girth were measured for all animals. Maternal postpartum mass (MPPM), percentage maternal expenditure (percentage of MPPM used by weaning), pup mass gain rate and weaning mass and percentage mass transfer efficiency (total mass gain of pup/ total mass loss of female × 100) were estimated (Pomeroy et al. 1999). We also recorded whether the mothers produced and weaned a pup on the Isle of May in the subsequent year (2014).

#### Foraging females

Foraging females were caught on or close to haul out sites, using hoop or seine nets (McConnell et al. 1999). Animals were weighed, and 0.5 mL 100 kg^−1^ intravenous Zoletil ^100^ was administered using 18-gauge, 3.5-in. spinal needles (BD systems) into the extradural vein (Bennett et al. 2017). All puncture areas were disinfected (Savlon: 3% w/v cetrimide, 0.3% w/v chlorhexidine gluconate; Novartis, Horsham, UK) before needle insertion and sprayed afterwards with Terramycin® (oxytetracycline, Pfizer Ltd.), as standard. Morphometric measurements were taken and a hind flipper tag (Dalton ID Systems, Henley on Thames, Oxon, UK) attached.

### Blubber biopsy procedure

One full-depth 6-mm biopsy (Acu-punch, Acuderm, Schuco International, Watford, UK) was taken from the dorso-lateral pelvic area under local anaesthesia (Lignol®, Dechra, Northwich, UK). A prophylactic intramuscular dose of antibiotic (Terramycin, 1 mL 10 kg^−1^; Pfizer Ltd.) was then injected. Biopsies were placed in RNase free cryogenic vials and immediately flash frozen and then stored at − 80 °C until analysis.

### RNA extraction and cDNA synthesis

Blubber (250–500 mg) was homogenised on ice in 1 mL TRIzol® Reagent (Ambion, Life Technologies, Paisley, UK) using a hand-held electric homogeniser (Disperser T10 basic ULTRA-TURRAX®, IKA®, Staufen, Germany). The middle section of the tissue biopsy, rather than inner (nearer the muscle) or outer (nearer the skin), was chosen to allow standardised use of all samples for which the orientation was not clear after freezing and to minimise possible differences due to variable tissue depth (Koopman 2007; Strandberg et al. 2008). RNA was extracted using TRIzol-chloroform (Sigma Aldrich Co., Dorset, UK). The pellet was air dried at room temperature for 5–10 min and resuspended in 15 μL molecular grade water (HyClone™, GE Healthcare Life Sciences, Hertfordshire, UK). RNA concentration and purity were measured using a NanoDrop 2000 Spectrophotometer (Thermo Scientific, Basingstoke, UK). RNA was diluted to 500 ng μL^−1^, and its integrity was determined by gel electrophoresis on a 1% agarose gel stained with SYBR® Safe (Invitrogen, Life Technologies, Paisley, UK). RNA was stored at − 80 °C before 250 ng was used for cDNA synthesis (QuantiTect Reverse Transcription kit; QIAgen, Manchester, UK), including a genomic DNA (gDNA) elimination step (42 °C for 2 min).

### Primer design and testing

Primer pairs (Tm of 60 °C) were designed using Primer3 software (Untergasser et al. 2012) against the conserved regions of a range of reference genes, REs and HSPs (Table 1) using sequences from Carnivore species (Phocidae; Otariidae; Odobenidae; Ursidae; Mustelidae; Canidae) from the National Centre for Biotechnology Information protein database (http://www.ncbi.nlm.nih.gov/protein) aligned using Clustal Omega (http://www.ebi.ac.uk/Tools/msa/clustalo/). Primers were synthesised by MWG Eurofins Operon (Ebersberg, Germany), Sigma-Aldrich (Dorset, UK) or Integrated DNA Technologies (Leuven, Belgium).

**Table 1:** Sequences for forward and reverse primers, amplicon size and efficiency of qRT-PCR reactions for each reference gene or gene of interest. ^Δ^Taken from Beineke et al. (2004); ^ΔΔ^taken from Tabuchi et al. (2006)

Gene	Primer sequence 5’-3’		Size	Efficiency (%)
	Forward	Reverse		
*YWHAZ*	GAGGTTGCTGCTGGTGATGA	TCCGGGGAGTTCAGAATTTCG	170	91.61
*L8*^*ΔΔ*^	GGTGTGGCTATGAATCCTGT	ACGACGAGCAGCAATAAGAC	126	113.26
*GAPDH*^*Δ*^	GCCAAAAGGGTCATCATCTC	GGGGCCATCCACAGTCTTCT	232	123.21
*CycA*	TCATCTGCACCGCCAAGAC	AAGCGCTCCATGGCTTCCAC	260	93.20
*S9*	ACATCCCGTCCTTCATTGTC	CAATCCTCCTCCTCGTCATC	157	101.44
*UXT*	CTCACAGAGCTCAGCGACAG	AGGTGTCTCCGGGAAATTCT	117	118.41
*Hsp70*	AAGATCACCATCACCAACGA	AAATCACCTCCTGGCACTTG	238	101.56
*Hsc70*	AATCAAGTTGCGATGAACCC	CCTGCCAGCATCATTCAC	139	102.90
*Hsp90*	TGGAGCGTCTTCGGAAG	TCTGGAAGTTCCAAGCCCT	139	99.24
*Hsp40*	CCTGGAATGGTTCAGCAAAT	GCCATCTTTCATGCCTTTGT	159	91.16
*Hsp27*	AGCTGACGGTCAAGACGAAG	GGCAGCGTGTATTTTCGAGT	112	97.32
*GPx*	TTGTCAACGTGGCCAGCTA	TGAGGCTGGGTAGGATTTCC	158	96.13
*CAT*	GGTAATTGGGATCTTGTTGG	ATGGTCTGGGACTTCTGG	140	101.42
*SOD*	CCTGGAGCCTCACATCAAC	TAGCTCTTCAGCCTGGGCT	127	98.63
*GST*	CCTCAAGGAGAGAACCCTGA	CTGGGCCATGTTAACCACTT	123	92.26
*Nox4*	AGCCTCCGCATCTGTTCTTA	CTTCTGGTTCTCCTGCTTGG	114	113.74

A blubber cDNA pool was the template used to amplify each target using Taq polymerase chain reaction (PCR) core kit (QIAgen, Manchester, UK) in a standard thermal cycler (Prime, Techne, Bibby Scientific, Staffordshire, UK: 94 °C for 30 s; 35 cycles of: 30 s at 94 °C, 30 s at 60 °C and 1 min at 72 °C; final extension: 72 °C for 1 min) to ensure each primer pair produced a single amplicon of the correct predicted size, verified on a 1% agarose gel. Those that produced a single amplicon were tested for amplification efficiency using quantitative real-time PCR (qRT-PCR) using a StepOne™ Real-Time PCR System (Applied Biosystems, Thermo Fisher, Loughborough, UK: 94 °C for 10 min; 40 cycles of: 95 °C for 15 s, 60 °C for 1 min; 95 °C for 15 s, followed by melt curve analysis).

Each primer pair was run in triplicate using a log serial dilution of pooled cDNA template, and efficiency was calculated using standard methods (Pfaffl 2001). Primer pairs with amplification efficiencies of between 90 and 110% were used in further analysis (Taylor et al. 2010). Endpoint PCR products were cleaned up using ExoSAP (Exo; #EN0581; Thermo Fisher Scientific™) and shrimp alkaline phosphatase (SAP; #EF0511; Fermentas Life Sciences, Thermo Fisher Scientific) and subject to blunt end cloning using calcium chloride transformation of competent *Escherichia coli* (CloneJET™ PCR Cloning kit, #K1231; Thermo Fisher Scientific). Sequences from positive colonies were verified using Sanger sequencing (Source BioScience Ltd, Nottingham, UK).

### Reference gene selection

*NormFinder* (Andersen et al. 2004; http://moma.dk/normfinder-software) and *BestKeeper* (Pfaffl et al. 2004) were used to identify stably expressed genes from cyclin A (*CycA*), glyceraldehyde 3-phosphate dehydrogenase (*GAPDH*), ribosomal protein S9 (*S9*), ribosomal protein L8 (*L8*), ubiquitously expressed prefoldin-like chaperone (*UXT*) and tyrosine 3-monooxygenase/tryptophan 5-monooxygenase activation protein zeta (*YWHAZ*). *CycA* and *S9* were identified as the most appropriate reference genes for this study.

### Relative mRNA abundance quantification using qPCR

We measured relative mRNA abundance in qRT-PCR using specific primers for genes of interest (GOI) including HSPs (*Hsp70*, *Hsc70*, *Hsp90*, *Hsp40*, *Hsp27*) and REs (*GPx*, *CAT*, *SOD*, *GST*, *Nox4*). No-template controls and reference genes *CycA* and *S9* were included on each plate. Molecular grade water was used to dilute cDNA 1:10 to produce template cDNA. The qRT-PCR was performed in either a 48-, 96- or 384-well plate with 4 μL of each template run in triplicate, using the StepOne system (Applied Biosystems) and 5 μL iTaq™ Universal SYBR® Green Supermix (Bio-Rad, Hertfordshire, UK) and forward and reverse primers at a concentration of 10 µM in each reaction. Data were inspected using StepOne™ Software version 2.3 (Applied Biosystems). Outliers within triplicates were removed. Mean threshold was calculated for each amplicon to standardise across all plates (Bennett et al. 2017). Baselines were comparable across all plates. Normalised values, used in subsequent statistical analyses and henceforth referred to as relative mRNA abundance, were calculated using Δ*C*_*T*_ (Livak and Schmittgen 2001).

### Malondialdehyde (MDA) concentrations

MDA concentration (nmol µL^−1^), an index of lipid peroxidation, was measured in duplicate (MAK085, Sigma-Aldrich, Dorset, UK) in an extract of ~ 10 mg of the blubber that remained after RNA extractions, according to manufacturer’s instructions. To eliminate turbidity, after homogenisation and centrifugation, the fat supernatant was bypassed and 200 µL infranatant was removed and filtered through a 0.20-µm, regenerated cellulose, 4-mm diameter syringe filter (Corning®, Corning Inc. Life Sciences, MA, USA). Only 14 mothers had sufficient blubber remaining for MDA measurements at both early and late lactations. There was insufficient tissue to measure lipid content.

### Statistical analysis

Statistical analyses were performed in R 4.2.1 (R Core Team 2022).

#### Effect of mass and lactation on blubber cellular defences and oxidative stress

A paired *t*-test was used to investigate the difference in mass and axial girth from mothers between early and late lactation. Correlations between relative mRNA abundance of each of the GOI (ΔC_*T*_) at both early and late lactation were explored using Spearman’s rank correlation, and outlier abundance values were identified for each GOI. We examined the change in relative mRNA abundance of each GOI and MDA levels, both with and without outlier females, using paired *t*-tests to determine whether they changed throughout lactation. The absolute change in relative mRNA abundance between early and late lactation was then calculated for each GOI. Clustering of the GOIs at both early and late lactation and their absolute changes during lactation were explored using principle component analyses (PCA) using the princomp function, to determine whether the variance in GOI data could be explained using fewer variables than all the GOIs individually. PCAs were conducted for all GOIs together and for the RE and HSP genes separately. In each case, the PCA was performed both with and without outlier females (6L, 5B and 0H) to explore the potential influence of extreme values for some GOI.

To determine whether initial resources dictated patterns of gene expression, general additive models (GAMs) from the ‘mgcv’ package (Wood 2011) were then used to investigate whether the principle components (PCs) of the relative mRNA abundance for the GOIs at early lactation were a function of maternal body mass at early lactation. Continuous covariates were fitted as smoothed terms with shrinkage, with the degree of smoothing defined manually as 3 knots (k). Where the response was linear, the data were reanalysed using a general linear model (GLM). Next, to investigate whether initial resources or the absolute change in maternal mass affected the change in GOI expression, GAMs or GLMs, as appropriate, were used to investigate whether the PCs of absolute change in relative mRNA abundance for the GOIs during lactation were a function of maternal mass at early lactation, relative change in maternal mass during lactation or an interaction between the two. Model fits were assessed by examination of residuals and qq plots. Outliers were identified, examined and removed when they were > 2 SD from the mean.

We hypothesised that gene expression level was associated with evidence of blubber peroxidation. Using MDA concentration as a proxy for blubber peroxidation, we then investigated whether the PCs for the GOI mRNA abundance at early and late lactation were correlated with MDA concentration at the respective time point.

#### Effect of cellular defences and oxidative stress on maternal performance

We then investigated the association between oxidative stress and/or the PCs of cellular defences and maternal performance. We performed linear regression (LM) for each of the following performance metrics as dependent variables: maternal mass loss rate, percentage maternal expenditure, pup mass gain rate, pup weaning mass and percentage mass transfer efficiency. Explanatory variables were the PCs 1–3 for the *Hsps* and *RE*s considered separately at early lactation, MDA concentration at early lactation and MPPM, a well-established predictor of maternal reproductive expenditure and pupping success (Pomeroy et al. 1999). For each model, co-linearity between explanatory variables was examined by calculating the variance inflation factor (vif), using the vif function from the ‘car’ package (Fox and Weisberg 2019), and only variables with vif > 3 were included (Zuur et al. 2010). Backwards model selection by corrected Akaike Information Criteria (AICc) score was then performed using the dredge function in the ‘MuMIn’ package (Barton 2018). Models identified by dredge where ΔAICc was < 2 were then assessed for model fit using residual and qq plots. The ranked model weights (AICc weight, the relative likelihood of the model) were also considered when determining the best overall model. Partial eta-squared was calculated for each LM using the etasq function from the ‘heplots’ package (Friendly et al. 2022). We used *t*-tests to investigate differences in blubber MDA concentrations and PC scores (PCs 1–3) for both *Hsps* and *REs*, considered separately at early and late lactation, and the PCs 1–3 of the absolute change in mRNA abundance of the GOIs during lactation between mothers that did and did not rear a pup in 2014 on the Isle of May, to explore whether blubber oxidative stress and/or mRNA abundance of the GOIs across lactation in 1 year was associated with apparent pupping success the following year.

#### Foraging and fasting differences in cellular defences and oxidative stress

It was not possible to capture the same individuals during both foraging and breeding. Samples from foraging seals were collected c. 400 km south of the breeding location. However, grey seals forage > 100 km from their haul out sites and move between haul out sites hundreds of kilometres apart (McConnell et al. 1999; Carter et al. 2022). Up to 60% of seals that feed predominantly in the region in which these foraging samples were taken breed in the region where these breeding samples were taken (Russell et al. 2013), suggesting the samples broadly represent the same population of animals. We sought to establish whether fasting-lactating mothers or foraging (presumed pregnant) females experienced higher defences and oxidative damage. We used PCA to investigate whether there was a difference in relative mRNA abundance of *Hsps* and *RE*s, considered separately, between early lactation and foraging (presumed pregnant) females. Relative mRNA abundance of all GOIs (Δ*C*_*T*_) was log_10_ transformed prior to the analyses to normalise the data. We also used *t*-tests to establish whether there was a difference in PC scores (PCs 1–3), mass or MDA concentrations between the two life history stages. Outliers in MDA were removed when values were > 2 SD from the mean.

## Results

### Female characteristics and maternal performance metrics

Mass (23.20% ± 4.71 (SD): paired *t*-test, *df* = 17, *T* = 20.52, *p* > 0.0001;* n* = 17) and axial girth (12.62% ± 4.42 (SD): paired *t*-test, *df* = 17, *T* = 11.43, *p* > 0.0001;* n* = 17) fell between early and late lactations (Table 2).

**Table 2:** Range and mean ± SD of maternal mass and performance characteristics for female grey seals (*n* = 18) from the Isle of May 2013. MPPM= maternal postpartum mass

Performance characteristic	Range	Mean ± SD
MPPM (kg)	169.6–269	198.40 ± 27.37
Lactation duration (days)	17–25	20.16 ± 2.01
Maternal mass loss rate (kg day^−1^)	2.92–5.32	3.73 ± 0.59
Percentage maternal expenditure (%)	29.93–47.11	37.63 ± 4.22
Pup weaning mass (kg)	35.56–61.09	48.83 ± 7.55

### mRNA abundance of blubber cellular defences and lipid peroxidation during lactation

**Fig. 1:**
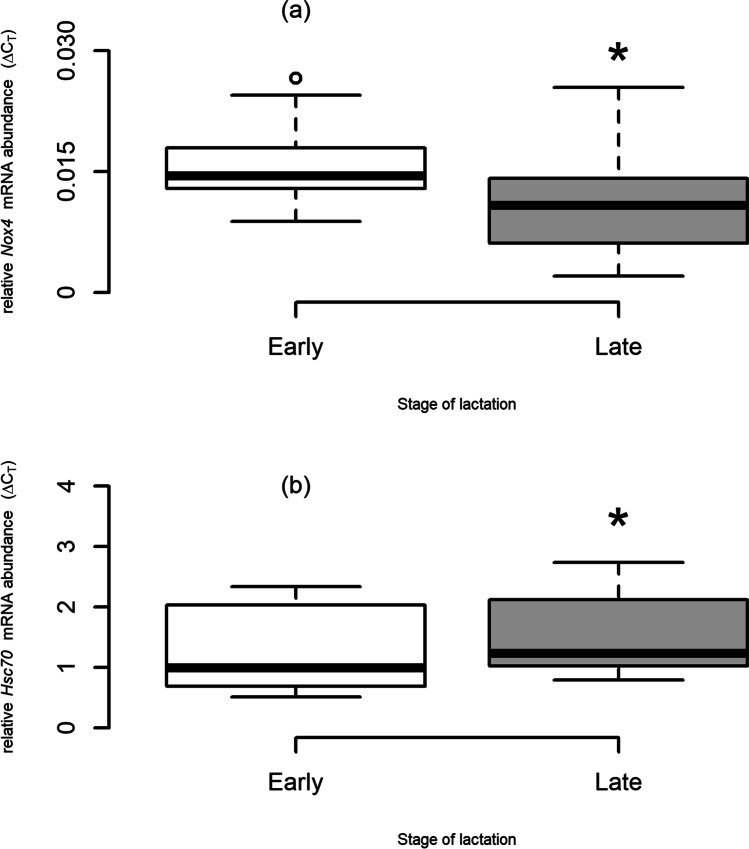
Change in relative mRNA abundance (calculated as ΔC_*T*_) of **a** *Nox4* (*n* = 17; all females) and **b** *Hsc70* (*n* = 14; outlier females removed) between early and late lactation in grey seal mothers. Boxes represent interquartile range (IQR), horizontal bar represents median, whiskers represent 1.5 times IQR and points represent outliers. Asterisk indicates significantly lower or higher values

Relative mRNA abundance of HSP genes in maternal blubber was mostly highly correlated with each other at both early and late lactations (*r*_s_ > 0.60; Supp Mat Fig. 1a and b). At early lactation, *Hsp70* and *Hsp27* were weakly correlated (*r*_s_ = 0.54; Supp Mat Fig. 1a). However, at late lactation, *Hsp27* mRNA abundance was unrelated to that of all other *Hsps* (*r*_s_ = 0.17–0.33; Supp Mat Fig. 1b). In addition, *Hsp70* and *Hsc70* were unrelated at late lactation (*r*_s_ = 0.16). In contrast, relative mRNA abundance of RE genes was weakly correlated or unrelated at early lactation (*r*_s_ = 0.056–0.36; Supp Mat Fig. 1a), with the exception of *GPx* and *SOD*, which were highly correlated (*r*_s_ = 0.72). At late lactation, abundance of RE genes became mostly highly correlated with each other (*r*_s_ > 0.60; Supp Mat Fig. 1b). *GPx* mRNA abundance was weakly correlated with *SOD* (*r*_s_ = 0.45) and *Nox4* (*r*_s_ = 0.38) and unrelated to *GST* (*r*_s_ = 0.10). *RE* and *Hsp* mRNA abundance were generally either not correlated or weakly correlated at both early and late lactation. However, at late lactation, *GST* was moderately correlated with both *Hsc70* and *Hsp40* (*r*_s_ =  − 0.49; − 0.51) and *Nox4* with *Hsp70* mRNA abundance (*r*_s_ =  − 0.54; Supp Mat Fig. 1b).

Relative mRNA abundance of *Nox4* was significantly lower during late lactation than early lactation (Fig. 1a; Table 3) when all females were considered. The difference was less pronounced when outliers were removed, but these females were not outliers for RE genes, suggesting a genuine reduction. Relative *Hsc70* abundance increased from early to late lactation (Fig. 1b; Table 3) when the outlier females were removed, but not when all females were considered. Mothers showed no change in relative mRNA abundance of any of the other RE or HSP genes (paired *t*-test, *p* > 0.05; *n* = 17; Table 3).

**Table 3:** Paired *t* test output for the change in each GOI from early to late lactation for all females (left; *df* = 16) and when the outliers for extreme *Hsp* values (5B, 6L and 0H) were removed (right; *df* = 13). Significant changes during lactation are highlighted in bold

GOI	All females			Outliers removed		
	Direction	*T*	*p*	Direction	*T*	*p*
*Hsp70*	-	1.35	0.196	-	0.14	0.894
*Hsc70*	-	1.58	0.135	**↑**	**2.41**	**0.031**
*Hsp90*	-	0.26	0.795	-	0.37	0.721
*Hsp40*	-	1.37	0.190	-	1.57	0.141
*Hsp27*	-	0.36	0.726	-	0.91	0.378
*GPx*	-	1.45	0.166	-	1.30	0.218
*CAT*	-	0.28	0.780	-	0.57	0.577
*SOD*	-	0.10	0.923	-	0.77	0.455
*GST*	-	0.32	0.753	-	0.33	0.746
*Nox4*	**↓**	**2.59**	**0.020**	-	2.12	0.053

At early lactation, PC1–3 accounted for 99.62% of the variance in relative mRNA abundance for all GOI combined, when all females were included and 98.77% when outliers were removed. PC1, which accounted for 96.49% of the variance, was explained by *Hsc70* and *Hsp90* when all females were included and was dominated by *Hsc70* (accounting for 93.34% of the variance) when outliers were removed (Supp Mat Table 1). PC2, which accounted for 2.29% and 3.52% of the variance, with and without outlier females respectively, was dominated by high transcript abundance of *Hsc70* and low transcript abundance of *Hsp90* when all females were considered, but with high transcript abundance of *Hsp90* when outliers were removed, showing a substantial influence of the small number of unusual females on PC2. In both cases, PC3 was dominated by *Hsp70* (Supp Mat Table 1).

When *Hsps* and *REs* were considered separately, PC1–3 *Hsps* accounted for 99.85% of the variance and PC1–3 *REs* accounted for 99.81%, with all females included. With outliers removed, PC1–3 *Hsps* accounted for 99.53% and PC1–3 *REs* 99.75% of the variance, in their respective PCAs.

In the PCA for *Hsps* at early lactation, the PCs were similar to that of the PCA for all GOI combined (Supp Mat Table 1). In the PCA for *REs* at early lactation, PC1 *REs* accounted for 61.89% and 53.04% of the variance with and without outliers, respectively. In both cases, *GPx* and *CAT* were almost equally dominant, *CAT* becoming more so with the removal of outliers. PC2 *REs* accounted for 29.59% and 38.56% of the variance with and without outliers, respectively, and was dominated by high *GPx* and low *CAT* abundance in each case; in contrast to PC1 *REs*, *CAT* loadings were negative. *GST* strongly dominated PC3 *REs* with and without outlier females, accounting for 8.32% and 8.16% of the variance, respectively.

Variance accounted for by (%) and loadings of PCs for the GOIs at late lactation PCA and absolute change in relative mRNA abundance of GOIs (from early to late lactation) PCA (PCs labelled as ∆*Hsps* and ∆*REs*) are reported in Supp Mat Tables 2a and b and 3a and b.

There were no associations between PC1 *Hsps* and PC1 *REs* at early lactation or PC1 ∆*Hsps* or PC1 ∆*REs* and mass of mothers at early lactation or absolute change in mass across the lactation period (GLM: *p* > 0.05).

MDA concentration did not differ between early (0.072 ± 0.024; *n* = 15) and late lactation (0.066 ± 0.022; *n* = 13; paired t-test, *df* = 13, *T* = 0.61, *p* = 0.55). MDA was not associated with initial body mass in GLM or correlated with PCs 1–3 of GOI mRNA abundance at early or late lactation (*r* = 0.07–0.34; GLM: *p* > 0.05).

### Relationship between blubber cellular defences, lipid peroxidation and performance

Model AICc weights are reported in Table 4. For each performance metric, the AICc weight of the best model was ≈ double the weight of the next-ranked model. All lactating-fasting mothers were included in the analyses. The rate of maternal mass loss was positively related to MPPM (Table 4). Other variables did not improve the model. Lactation duration was positively related to PC2 for *Hsps* and PC2 for *RE*s at early lactation (Table 4; Supp Mat Table 1), such that females with higher initial blubber *Hsc70* and *GPx* and lower *Hsp90* and *CAT* expression undertook longer lactation periods. Maternal mass transfer efficiency was positively related to lactation duration and negatively related to PC2 *REs* at early lactation, such that, given the effect of longer suckling periods, mothers with higher initial blubber *GPx* and lower *CAT* expression were less efficient at transferring mass to their pups. Pup mass gain rate was positively related to both maternal mass loss rate and maternal efficiency of transfer, as expected. In addition, it was negatively associated with PC3 *REs* at early lactation (Supp Mat Table 1), such that pups of mothers with higher initial blubber *GST* expression gained mass more slowly. Pup weaning mass was positively related to lactation duration and had a negative relationship with PC2 *REs* at early lactation (Supp Mat Table 1), suggesting that, once lactation duration was accounted for, mothers with higher initial blubber expression of *GPx* and lower *CAT* produced smaller pups.

**Table 4:** Model output for linear models (LMs) using principle component (PC) scores of normalised gene expression of redox enzymes (*REs*) and *Hsps* at early lactation and appropriate maternal performance metrics to explain variability in rate of mass loss, lactation duration, pup weaning mass, pup mass gain rate and maternal mass transfer efficiency in grey seals

Performance metric	Covariate	Parameter estimate	*T* value	*p* value	Partial eta squared (η^2^_*p*_)	AICc weight
Maternal rate of mass loss (kg day^−1^)	MPPM	0.012	2.76	0.015	0.35	0.182
Lactation duration (days)	PC2* Hsps*	3.76	2.62	0.021	0.35	0.212
	PC2* REs*	18.24	2.28	0.041	0.28	
Pup weaning mass (kg)	PC2* REs*	− 50.29	− 2.28	0.042	0.30	0.247
	Lactation duration	2.87	4.73	< 0.001	0.65	
	Maternal rate of mass loss	7.05	3.66	0.003	0.53	
Pup mass gain rate (kg day^−1^)	Maternal rate of mass loss	0.41	3.29	< 0.001	0.47	0.198
	Efficiency of mass transfer (%)	0.016	2.84	0.015	0.40	
	PC3* REs*	− 7.90	− 2.21	0.047	0.29	
Maternal mass transfer efficiency (%)	Lactation duration	3.46	2.72	0.017	0.36	0.351
	PC2* REs*	− 208.80	− 4.37	< 0.001	0.59	

MDA concentration (Student’s *t*-test, *df* = 10, *T* = 1.25, *p* = 0.12; *n* (pupped 2013 only) = 9; *n* (pupped 2013 and 2014) = 9) and the PC (1–3) scores of *Hsps* and *REs* at early and late lactation and absolute change in GOIs (Student’s *t*-tests, *p* > 0.05; *n* (pupped 2013 only) = 9; *n* (pupped 2013 and 2014) = 8) did not differ between mothers that were only observed to pup in 2013 and those that also produced a pup in the following year on the Isle of May.

### Comparison between foraging and lactating-fasting females

Foraging female body mass was 117.4 ± 16.3 kg (SD; range = 80.8 kg to 150.4 kg) and axial girth was 128.8 ± 6.2 cm (SD: range = 127–141 cm). Early lactation mothers were significantly heavier (Student’s *t*-test, *df* = 29, *T* = 6.92, *p* < 0.001; *n* (lactating) = 17; *n* (foraging) = 13) and had greater girth (Student’s *t*-test, *df* = 29, *T* = 5.99, *p* < 0.0001; *n* (lactating) = 18; *n* (foraging) = 13) than foraging conspecifics.

**Fig. 2:**
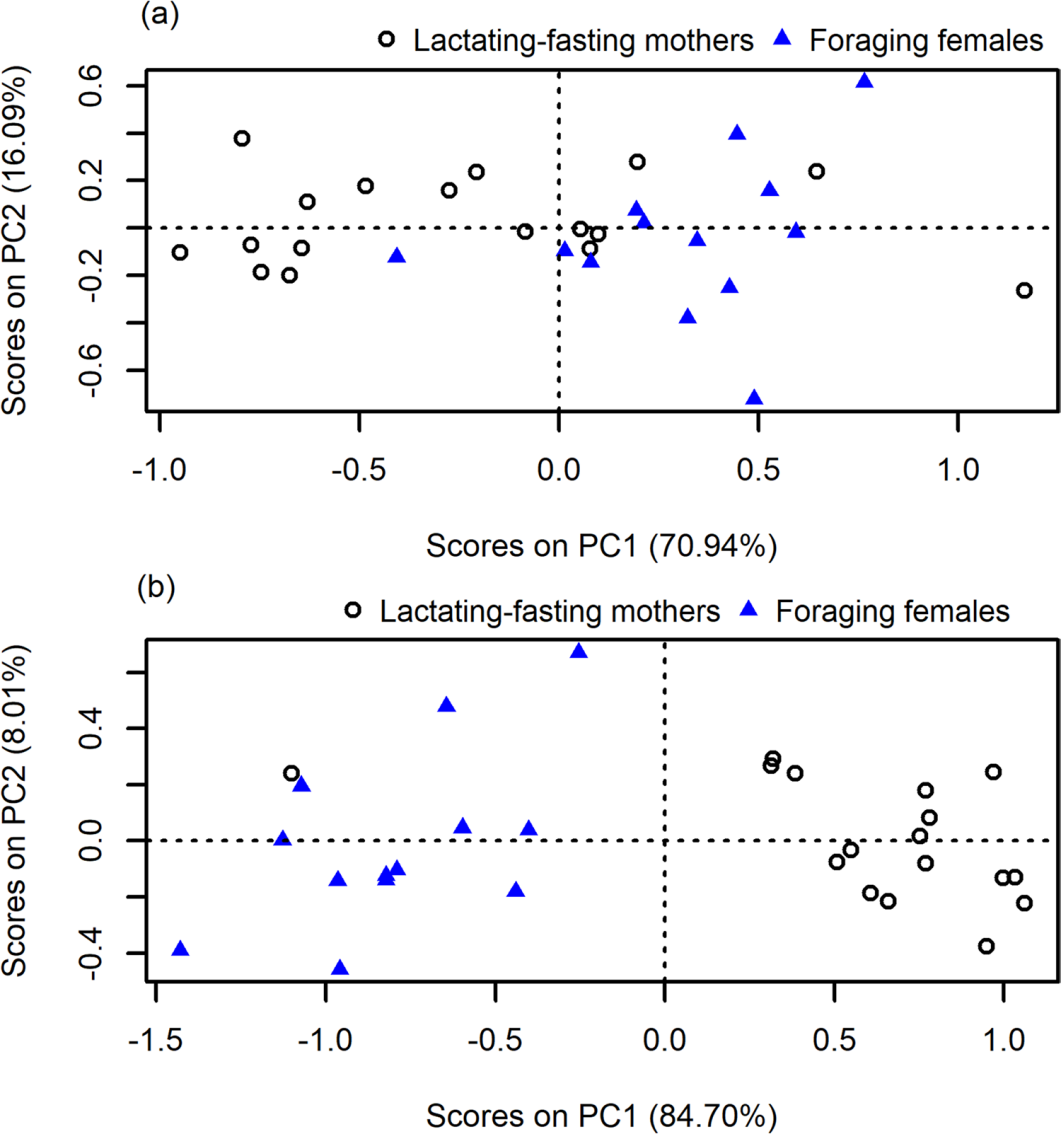
Principle component (PC) scores of relative mRNA abundance (calculated as ΔC_*T*_) of **a** *Hsp*s and **b** *RE*s in female grey seals at early lactation (black, open circle; lactating-fasting mothers; *n* = 17) and during the summer foraging period (blue, closed triangle; foraging (presumed pregnant) females; *n* = 13). All females were included. Percentage of total variance explained by the two principal components is indicated (%)

During foraging (and thus presumed pregnancy), blubber relative mRNA abundance of *Hsc70* was highly correlated with *Hsp90*, *Hsp40*, *GPx* and *CAT* (*r*_s_ > 0.60; Supp Mat Fig. 2). *Hsp90* was strongly correlated with *Hsp40* (*r*_s_ = 0.63) and *Hsp27* (*r*_s_ = 0.70), and *Hsp40* was very strongly related to *CAT* mRNA abundance (*r*_s_ =  − 0.94). Relative mRNA abundance of RE genes was either not correlated or weakly correlated (*r*_s_ = 0.003–0.49; Supp Mat Fig. 2). *Hsp70*, *SOD*, *GST* and *Nox4* were weakly correlated with all other GOIs during foraging (*r*_s_ =  − 0.004 to 0.49; Supp Mat Fig. 2).

PC (1–2) scores of lactating-fasting mothers clustered very differently from the foraging (presumed pregnant) females, for both *Hsps* and *REs* (Fig. 2 a and b). PC1 *Hsps*, which accounted for 70.94% of the variance, was mostly dominated by log_10_ *Hsp90* and log_10_ *Hsp27*, which contributed almost equally to PC1 (Supp Mat Table 4). PC1 *Hsps* scores were significantly lower in lactating-fasting mothers, suggesting higher mRNA abundance of log_10_ *Hsp90* and log_10_ *Hsp27* in foraging females (*t*-test: *p* < 0.05; Fig. 2a). PC2 *Hsps*, which accounted for 16.09% of the variance, was dominated by log_10_ *Hsp70* (Supp Mat Table 4). There was no difference in PC2 *Hsps* scores (*t*-test: *p* > 0.05), which were generally low (Fig. 2a), between foraging females and fasting-lactating mothers, suggesting log_10_ *Hsp70* did not differ between the reproductive states.

**Fig. 3:**
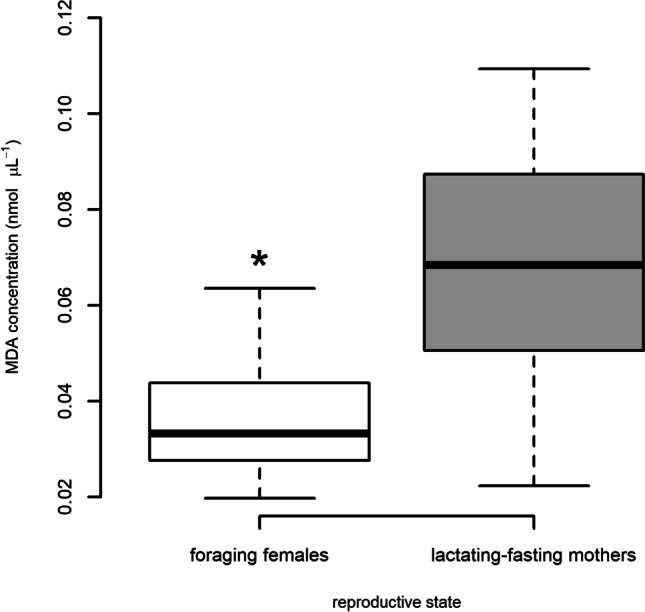
Difference in MDA concentrations (nmol µL^−1^) between foraging (presumed pregnant; *n* = 12) and lactating-fasting (*n* = 16) female grey seals. Boxes represent interquartile range (IQR), horizontal bar represents median, whiskers represent 1.5 times IQR and points represent outliers. Asterisk indicates significantly different values (*t*-test; *p* < 0.05)

An initial screening of the relative *RE* mRNA abundance indicated that, when considered individually, all REs were lower in blubber of foraging (presumed pregnant) females, compared to lactating-fasting mothers (Supp Mat Fig. 3). This was supported by the PCA, as all foraging (presumed pregnant) individuals were negatively associated with PC1 *REs*. PC1 *REs*, which accounted for 84.70% of the variance, were dominated by log_10_ *GPx* (Supp Mat Table 2). All relative *RE* mRNA abundance PC1 scores were significantly lower in foraging females, compared to lactating-fasting mothers (*t*-test: *p* < 0.05; Fig. 2b), suggesting a higher mRNA abundance of *GPx* in breeding individuals. PC2 *REs*, which accounted for 8.01% of the variance, were dominated by log_10_ *GST* mRNA abundance (Supp Mat Table 2). There was no significant difference in PC2 *REs* scores between females during foraging (t-test: *p* > 0.05) and lactating-fasting mothers (*t*-test: *p* > 0.05).

Foraging females had significantly lower blubber MDA than lactating-fasting mothers (*t*-test, *df* = 26, *T* = 5.76, *p* < 0.0001; *n* (lactating-fasting mothers) = 16; *n* (foraging (presumed pregnant)) = 12; Fig. 3).

## Discussion

Most mammals increase food intake and absorption efficiency during lactation to support increased energetic costs (Cripps and Williams 1975), but may still experience caloric deficit, resulting in resource limitation (Prentice and Prentice 1988). Under such circumstances, allocation of resources to cellular defences is predicted to be lower and oxidative damage greater (Costantini 2008; Garrett et al. 2011; Speakman et al. 2015; Al Jothery et al. 2016). Our data from capital breeding grey seals, which naturally accrue substantial fat depots prior to breeding, but may nevertheless experience resource limitation during lactation, appear to support this hypothesis. The pattern of expression of a number of genes associated with cellular defences was substantially different, with redox enzyme expression lower and levels of one marker of lipid peroxidation higher in blubber from fasting, lactating mothers compared to presumed pregnant, foraging females. Lactation duration was positively associated with expression of a number of cellular defence genes, particularly high abundance of the antioxidant enzyme *GPx* but low abundance of *CAT*. We suggest that maternal cellular stress in blubber may influence lactation duration, pup mass gain and maternal mass transfer efficiency. The ability to mount effective cellular defences in blubber may mitigate against impacts of oxidative stress on lactation duration, but maintaining antioxidant defences could impede maternal transfer efficiency with consequences for maternal performance and, potentially, pup mass and survival. Understanding the role of cellular stress in lactation strategy and young-rearing performance has wider relevance to other species that are likely to experience similar trade-offs because they fast during highly energy demanding activities, such as migration and/or provisioning of young. It is also important to establish whether additional, environmental sources of stress may compound existing trade-offs, with fitness consequences for mothers or offspring, in an increasingly exploited, polluted and warming environment.

### Relative abundance of cellular defence mRNA and lipid peroxidation during lactation

Larger mothers did not have greater defences: body mass at the start of lactation was not associated with higher investment in blubber molecular chaperones and oxidative defences, which does not support the predictions of the life-history-oxidative stress theory. This highlights the need to understand the functional consequences of increased blubber expression of the GOI measured here, which may change for reasons other than cellular stress. The increase in blubber abundance of *Hsc70* mRNA during lactation in most females suggest allocation to production of chaperones typically occurs as fasting progresses. However, some females did not experience the *Hsc70* increase. The observed changes in *Hsc70* mRNA abundance over lactation, differences in *Hsp* profiles between females and differences between pregnancy and lactation may be more functionally relevant for sex steroid and glucocorticoid signalling than cellular stress (Pratt and Toft 2003; Razandi et al. 2010; Li et al. 2018).

Blubber *Nox4* was downregulated from early to late lactation, which should reduce RS production (Lambeth 2004), but oxidative damage, inferred from stable MDA concentration, was not ameliorated. Instead, since *Nox4* is a hallmark of adipocyte differentiation (Mouche et al. 2007; Schröder et al. 2009), the fall in its mRNA abundance in late lactation could indicate fewer mitotic pre-adipocytes relative to mature adipocytes. Lower *Nox4* in late lactation may also reflect decreased insulin sensitivity (Mahadev et al. 2004), which occurs in fasting, lactating elephant seals (Fowler et al. 2008). Measurements of RE activity and proteins involved in cellular defences would help to confirm the functional consequences of our gene expression data for cellular stress, but we had insufficient tissue to perform these assays.

The lack of change in relative mRNA abundance of the other HSPs and REs or in MDA levels during lactation, despite radical changes in body mass and axial girth, is similar to the maintenance of oxidative status during fasting in other capital breeders (Colominas-Ciuró et al. 2017), including female elephant seals (Sharick et al. 2015) and may indicate grey seal mothers do not experience increasing blubber oxidative or proteotoxic stress over the lactation fast. *Hsc70*, *Hsp90*, *GPx*, *CAT* and *GST* explained the majority of the variation in the mRNA abundance data in maternal blubber and could therefore be considered useful transcript-level cellular defence markers during lactation. However, our data reflect only a small selection of cellular defence genes and no specific proteotoxic stress markers. A wider suite of oxidative stress markers, such as protein carbonylation and other lipid peroxidation measures, would allow this hypothesis to be tested further, which would require more tissue than was obtained for this study.

### Performance during lactation is associated with blubber cellular defence status

Grey seal mothers appear to trade off pup-rearing against blubber cellular defences and redox status. Animals with higher mRNA abundance of *Hsc70* and lower *Hsp90* and higher *GPx* and lower *CAT* sustained a longer lactation, perhaps through mitigating blubber proteotoxic and oxidative stress. However, those with higher *GPx* and lower *CAT* also had lower mass transfer efficiency and produced pups with lower weaning mass. Additionally, pups of mothers with high *GST* mRNA abundance gained mass more slowly. We speculate that blubber cellular stress could modulate lactation duration or maternal investment capacity in these animals, which may limit ongoing damage to balance current and future reproductive fitness. It would be informative to explore other stress markers, examine whether proteotoxic and oxidative stress occurs in other tissues and has a similar association with lactation duration or maternal transfer efficiency and pup weaning mass. It is possible that other tissues, such as brain, are better protected, as observed in other species in which cellular defence investment and oxidative damage are tissue specific (Zhao et al. 2015). The role of RS in lipolysis may make blubber particularly vulnerable to oxidative stress during lactation (Krawczyk et al. 2012; Abou-Rjeileh and Contreras 2021).

Pup weaning mass, a key driver of first year survival in grey seals (Hall et al. 2001), was strongly positively related to lactation duration and maternal mass loss rate. Females that maintained high constitutive *Hsps* and *GPx* and low *CAT* were also able to undertake a longer lactation, suggesting an important link between defences and maternal performance. However, transfer efficiency was reduced in those with high *GPx* and low *CAT*, suggesting a trade-off between investment in pup rearing and in antioxidant defences. This needs to be confirmed by exploration of other oxidative stress markers and measurement of protein levels and activity of antioxidant enzymes. Higher maternal cellular stress in blubber may impact pup weaning mass, with potential consequences for first-year survival, if it can shorten lactation duration and/ or slow pup mass gain rate or transfer efficiency. Environmental stressors that exacerbate this cellular stress may thus have survival consequences for offspring.

There was no evidence that mothers with higher levels of MDA or cellular defence genes experienced reduced pupping success in the following year. Higher levels of MDA or differential investment in blubber cellular defences seen here therefore cannot explain why females often ‘skip’ breeding years (Pomeroy et al. 1999). While short-term cellular stress in key tissues could constrain behavioural choices in lactating mothers within one breeding season, lipid peroxidation is reversible (Sevanian et al. 1983; Fisher et al. 2018) such that transient oxidative stress in blubber associated with a single reproduction event does not appear to compromise adult female fitness in a future year, similar to findings in other animals (Pap et al. 2018). However, we cannot eliminate the possibility that cumulative stress or damage over many years hastens reproductive senescence or impacts on successful pup rearing rather than on pup production in future years. Additional markers of oxidative stress along with indicators of biological versus chronological age and repeat sampling from the same individuals in successive years to examine pupping and rearing to weaning would help examine this hypothesis.

### Comparison between foraging and lactating-fasting females

The current findings compare lactation with pregnancy because it is likely that most of the foraging females here were pregnant adults, based on nose-tail length and age at recruitment and fecundity estimates (Thomas et al. 2019). Indeed, three of the foraging females were observed with pups during the following breeding season. Foraging females had significantly lower relative *RE* mRNA abundance and MDA levels in blubber compared to lactating individuals. We cannot rule out that this difference is a result of fatter animals experiencing greater lipid peroxidation (Pérez-Rodríguez et al. 2015) because we were unable to correct for lipid content of the blubber tissue. However, if changes in MDA levels were purely a result of greater lipid supply, we would expect that larger mothers at the start of lactation would have higher MDA and that MDA levels would fall during fasting as animals lost condition, which did not occur. Female grey seals may thus be more vulnerable to blubber oxidative damage during the lactation fast than they are while pregnant and foraging, despite greater investment in RE during the lactation fast. Higher oxidative stress during young rearing is similar to findings in some other vertebrates (Alonso-Alvarez et al. 2004; Wiersma et al. 2004; Sawecki et al. 2019), but contrasts with greater oxidative imbalance during incubation than chick rearing in penguins (Colominas-Ciuró et al. 2017). Higher *RE* transcript abundance and MDA levels in the fatter and heavier females at early lactation compared to pregnant foraging females could reflect greater cellular stress in blubber that has larger adipocytes, and perhaps reduced vascularity, and thus slower oxygen delivery, compared to the foraging animals, which were smaller. Blubber pO_2_ in juvenile grey seals is within the normal range for WAT seen in other mammals, but inversely related to body fatness and tends to decrease as animals gain weight (Oller et al. 2021). Adult females are likely to be fatter than juveniles and may have less blubber vasculature, since they are no longer actively growing, and may thus experience lower pO_2_. Our data are also consistent with induction of oxidant production at the onset of fasting and/ or lactation (Sorensen et al. 2006; Garrett et al. 2011; Sharick et al. 2015; Al Jothery et al. 2016) when the need for RS to maintain lipolysis may be required. The increase in cellular defences and oxidative stress must have occurred either late in the foraging period, or before day 6 of lactation, consistent with rapid increases in milk production and fat content (Mellish et al. 1999), since we observed no changes between early and late lactation.

Higher relative *Hsp* abundance in foraging females may reflect the ability to allocate more resource to cellular defence during feeding. Greater proteotoxic stress and HSP induction could also arise as a result of fat expansion and associated inflammation (Grimsrud et al. 2007). Although other seal species seem able to avoid or minimise inflammation (Vázquez-Medina et al. 2010, 2011; Martinez et al. 2018), animals are rarely sampled to examine these markers during feeding phases of their life history. *Hsp90* involvement in sterol regulatory element-binding protein regulation (SREBPs; Kuan et al. 2017), regulation of steroid receptors by Hsps (Pratt and Toft 2003; Razandi et al. 2010; Li et al. 2018) or chaperoning of newly synthesised proteins during adipogenesis (Pechan 1991) may also explain the observed higher levels in foraging versus fasting individuals. Alternatively, *Hsp* and *RE* expression changes may be linked to fluctuating contaminant levels in blubber during weight gain and loss (Louis et al. 2014).

## Conclusion

Grey seal mothers appear to experience greater blubber oxidative damage to lipids during their lactation fast compared with levels seen during foraging when animals are fattening and presumed to be pregnant. These data suggest female grey seals prioritise pup-rearing at the expense of maintenance of adequate blubber cellular defences, despite higher expression of antioxidant genes during the lactation fast than when foraging. In turn, greater mRNA abundance of constitutive *Hsps* and *GPx* and lower *CAT* are associated with longer lactation. Females may trade off antioxidant gene expression against mass transfer efficiency to pup, but compensate, if possible, by extending lactation.

These data thus support the life-history-oxidative stress theory and suggest cellular stress in blubber could influence lactation strategy in capital breeders. This suggestion can be explored further by investigating other tissues, a wider suite of markers of both oxidative and proteotoxic stress, measures of enzyme activity and protein levels of key cellular defences as well as fat content and other contributors to local cellular stress in blubber, such as oxygen levels and contaminant concentrations. Our findings highlight the need to examine multiple life history stages to interpret stress markers and demonstrate that lactation during resource limitation is a period of heightened vulnerability, even for species that accrue resource prior to breeding. Understanding cellular stress constraints on key life history decisions is of particular importance for individuals, populations and species that fast during energetically demanding life stages and are exposed to additional natural or anthropogenic stressors in the wild, such as high pollutant loads and rapid climate change.

## Supplementary Information

Below is the link to the electronic supplementary material.

Supplementary file1 (DOCX 1.20 MB)

## Data Availability

These data are available through Figshare (current private link for review: https://figshare.com/s/8d2cdd0b574a7e2c205a).

## Declarations

### Disclaimer

The sponsors and funders had no part in the design, execution, analysis or writing of the study.
