# Supplementary material for: Fitness correlates of blubber oxidative stress and cellular defences in grey seals (Halichoerus grypus): support for the life-history-oxidative stress theory from an animal model of simultaneous lactation and fasting
Source: Cell Stress Chaperones. 2023 Mar 18;28(5):551–66. doi: 10.1007/s12192-023-01332-1 (PMC10469160; doi:10.1007/s12192-023-01332-1)
Supplement: Supplementary file 1 — Supplementary file1 (DOCX 1.20 MB) [file 12192_2023_1332_MOESM1_ESM.docx]

**Fitness correlates of blubber oxidative stress and cellular defences in grey seals (*Halichoerus grypus*): support for the life-history-oxidative stress theory from an animal model of simultaneous lactation and fasting**

**Cell Stress and Chaperones**

Holly C. Armstrong^1,2*^, Debbie J.F. Russell^3^, Simon E.W. Moss^3^, Paddy Pomeroy^3^, Kimberley A. Bennett^4^

1 Marine Biology and Ecology Research Centre, Plymouth University, Drake Circus, Plymouth, PL4 8AA, UK

2 School of Psychology and Neuroscience, University of St Andrews, KY16 9JP, UK

3 Sea Mammal Research Unit, Scottish Oceans Institute, University of St Andrews, KY16 8LB, UK

4 Division of Health Science, School of Applied Sciences, Abertay University, Dundee, DD1 1HG, UK

* corresponding author ha54@st-andrews.ac.uk

**Supplementary Materials**

**Figures**


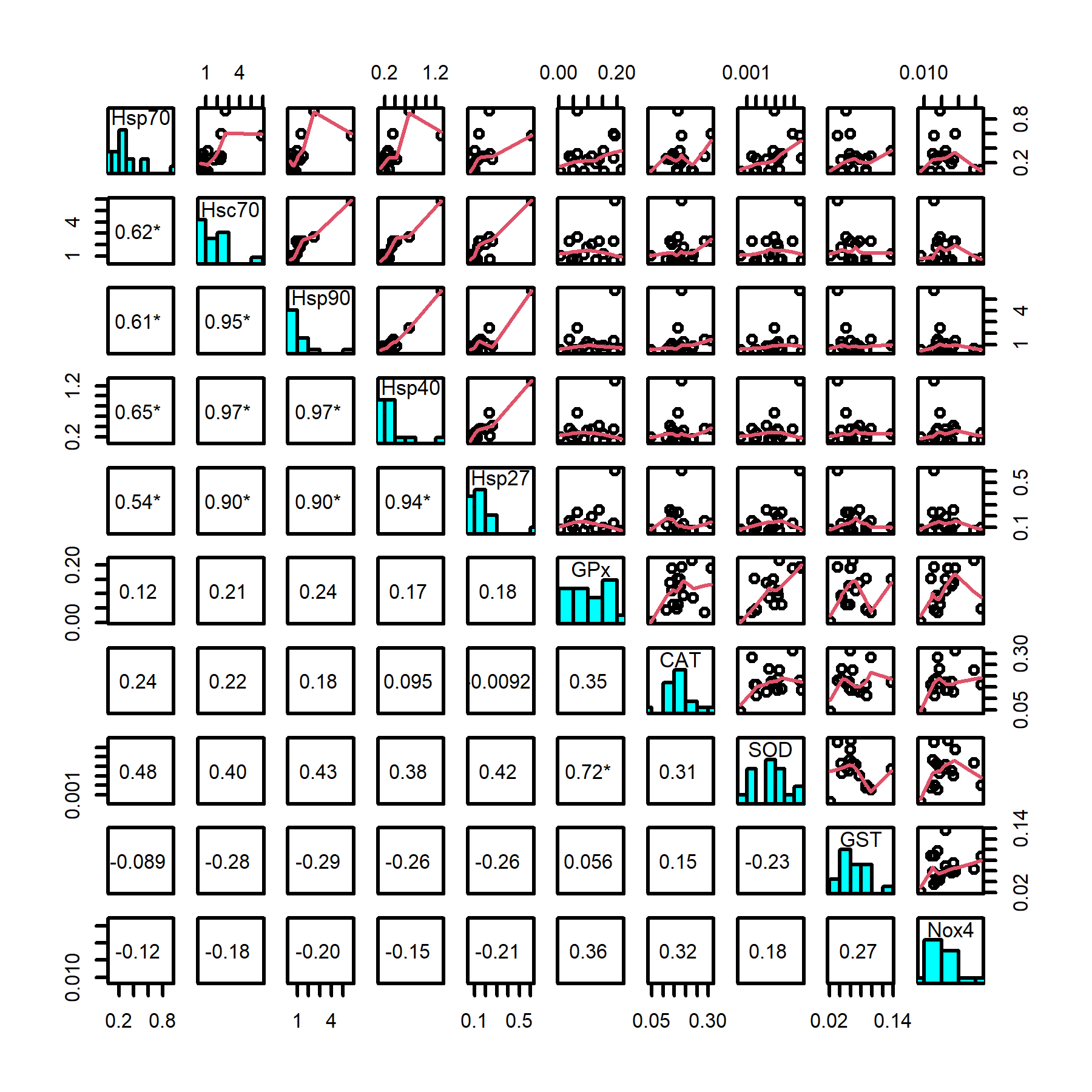


**a)**


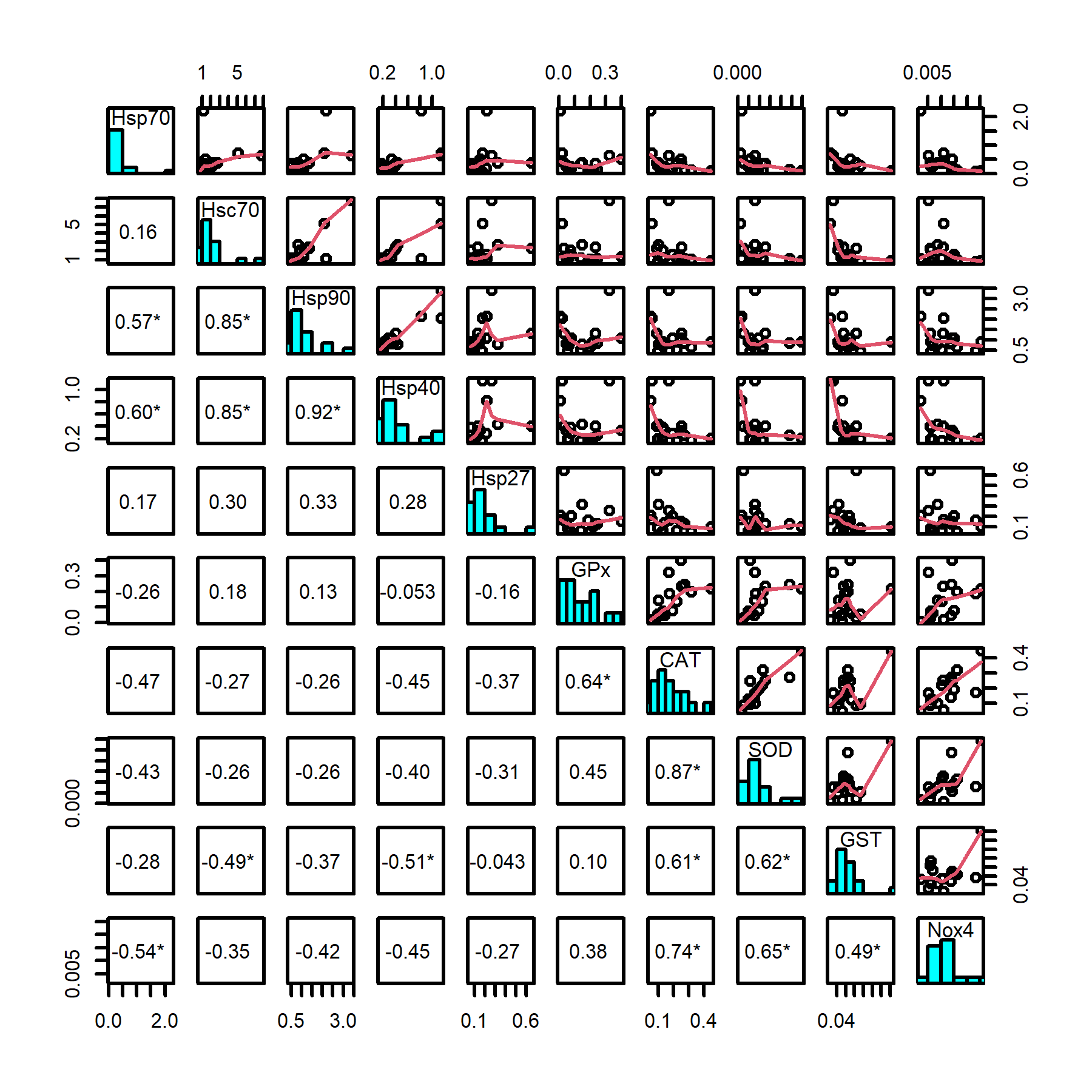


**b)**

**Supp Mat Fig 1** Spearman’s rank correlation between (left) and data distributions of (centre, blue bars; right) relative mRNA abundance of *Hsp*s and *RE*s in blubber from female grey seals during **a** early lactation (*n* = 17) and **b** late lactation (*n* = 17). * indicates a significant correlation (*p* < 0.05).


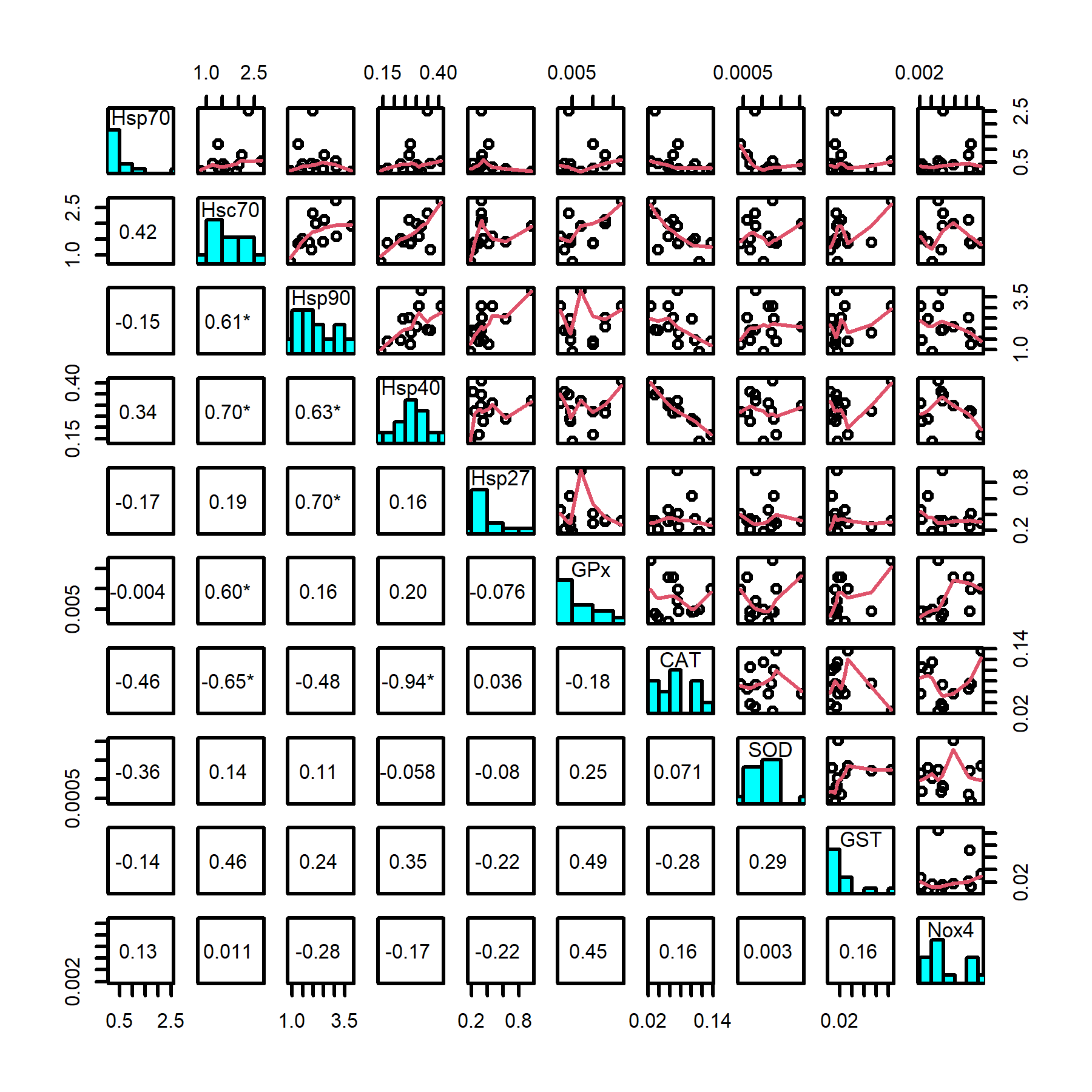


**Supp Mat Fig 2** Spearman’s rank correlation between (left) and data distributions of (centre, blue bars; right) relative mRNA abundance of *Hsps* and *RE*s in blubber from foraging (presumed pregnant) female grey seals (*n* = 13). * indicates a significant correlation (*p* < 0.05).


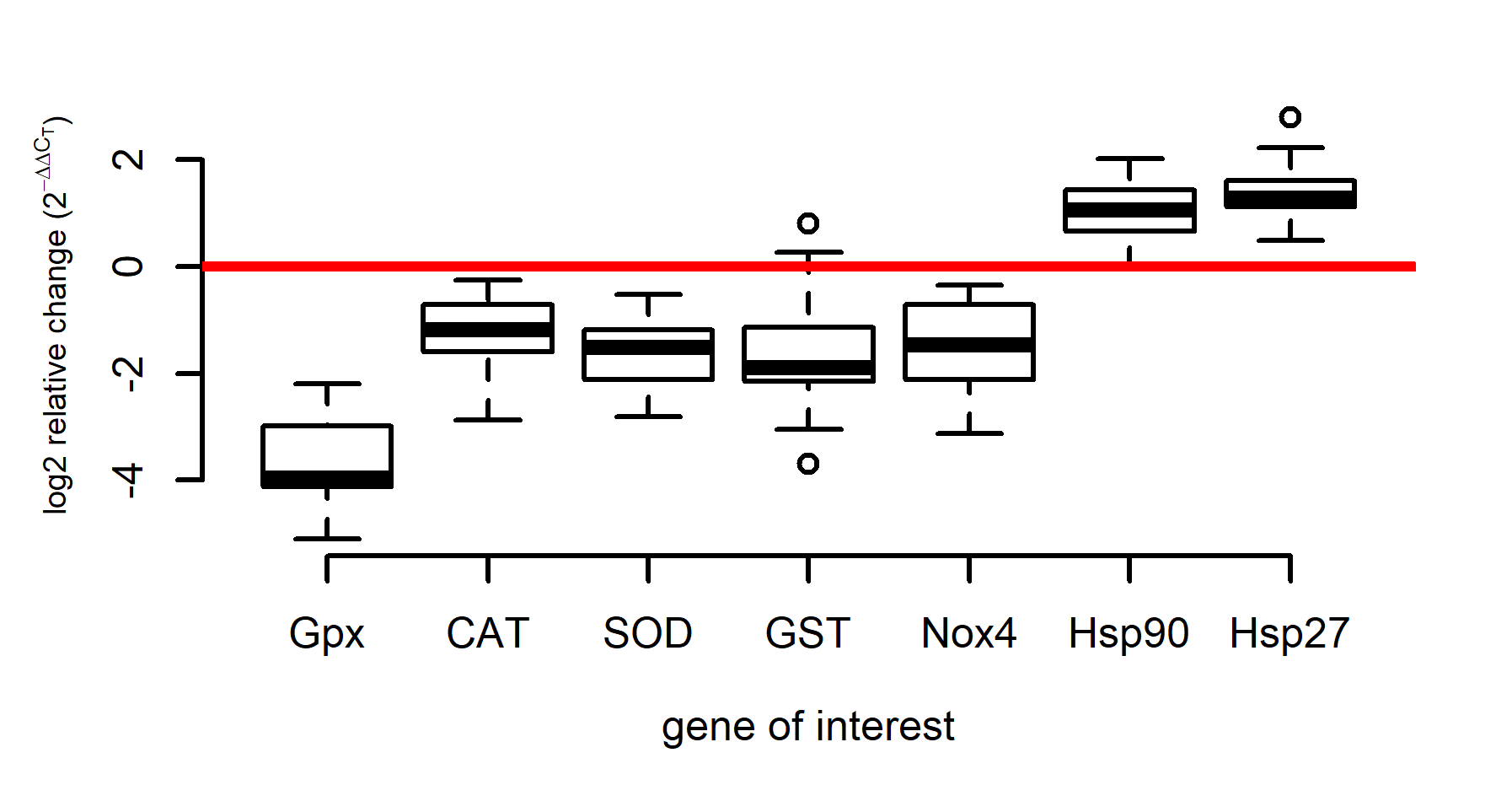


**Supp Mat Fig 3** mRNA abundance (calculated as ΔC*_T_*) differences in foraging (presumed pregnant) grey seal females relative to fasting-lactating mothers (T-test: *p* < 0.05). Boxes represent interquartile range (IQR), horizontal bar represents median, whiskers represent 1.5 x IQR and points represent outliers. Red line indicates parity between states.

**Tables**

|  | All females | | | Outliers removed | | |
| --- | --- | --- | --- | --- | --- | --- |
| Gene of Interest | PC1 | PC2 | PC3 | PC1 | PC2 | PC3 |
| *Hsp70* | 0.075 | 0.03 | **0.99** | 0.10 | 0.07 | **0.97** |
| *Hsc70* | **0.71** | **0.69** | -0.08 | **0.90** | -0.36 | -0.07 |
| *Hsp90* | **0.68** | **-0.72** | -0.03 | 0.40 | **0.85** | -0.09 |
| *Hsp40* | 0.15 | -0.03 | 0.07 | 0.11 | -0.18 | < -0.01 |
| *Hsp27* | 0.065 | < -0.01 | -0.04 | 0.04 | -0.22 | 0.08 |
| *GPx* | < 0.01 | -0.02 | < -0.01 | < -0.01 | 0.01 | 0.16 |
| *CAT* | < 0.01 | 0.03 | 0.05 | 0.04 | 0.24 | < 0.01 |
| *SOD* | < 0.01 | < -0.01 | < 0.01 | < 0.01 | < -0.01 | < 0.01 |
| *GST* | < -0.01 | < 0.01 | 0.02 | < 0.01 | < 0.01 | 0.03 |
| *Nox4* | < -0.01 | < 0.01 | < 0.01 | < -0.01 | < -0.01 | < -0.01 |
|  | PC1 *Hsps* | PC2 *Hsps* | PC3 *Hsps* | PC1 *Hsps* | PC2 *Hsps* | PC3 *Hsps* |
| Proportion of variance explained | 96.72 | 2.29 | 0.83 | 94.27 | 3.36 | 1.90 |
| Cumulative proportion of variance explained | 96.72 | 99.01 | 99.84 | 94.27 | 97.63 | 99.53 |
| Gene of Interest | PC1 *Hsps* | PC2 *Hsps* | PC3 *Hsps* | PC1 *Hsps* | PC2 *Hsps* | PC3 *Hsps* |
| *Hsp70* | 0.08 | 0.03 | **0.99** | 0.10 | 0.07 | **0.99** |
| *Hsc70* | **0.71** | **0.70** | -0.08 | **0.90** | -0.37 | -0.07 |
| *Hsp90* | **0.68** | **-0.72** | -0.04 | 0.40 | **0.88** | -0.08 |
| *Hsp40* | 0.15 | -0.03 | 0.07 | 0.11 | -0.17 | < 0.01 |
| *Hsp27* | 0.07 | < -0.01 | -0.03 | 0.04 | -0.22 | 0.10 |
| Gene of Interest | PC1 *REs* | PC2 *REs* | PC3 *REs* | PC1 *REs* | PC2 *REs* | PC3 *REs* |
| *GPx* | **0.71** | **0.70** | < 0.01 | **0.67** | **0.74** | 0.05 |
| *CAT* | **0.70** | **-0.71** | -0.09 | **0.74** | **-0.67** | 0.06 |
| *SOD* | 0.02 | 0.01 | -0.02 | 0.01 | 0.01 | -0.02 |
| *GST* | 0.06 | -0.07 | **0.99** | -0.07 | < 0.01 | **0.99** |
| *Nox4* | 0.03 | < 0.01 | 0.04 | 0.02 | 0.01 | 0.01 |

**Supp Mat Table 1** Loadings of Principle Components (PCs) 1-3 all GOI combined; *Hsps* and *REs* considered separately (early lactation). Proportion of variance explained by PCs are presented for the PCA for *Hsps* only. PCs are displayed for analyses including all females (*n* = 17) and when the outliers for extreme *Hsp* values (5B, 6L and 0H) were removed (*n* = 14).

**Supp Mat Table 2** Proportion of variance explained by and loadings of PCs 1-3 all GOI combined; *Hsps* and *REs* considered separately (late lactation). PCs are displayed for analyses including all females (*n* = 17) and when the outliers for extreme *Hsp* values (5B, 6L and 0H) were removed (*n* = 14).

|  | All females | | | Outliers removed | | |
| --- | --- | --- | --- | --- | --- | --- |
|  | PC1 | PC2 | PC3 | PC1 | PC2 | PC3 |
| Proportion of variance explained | 0.89 | 0.08 | 0.01 | 0.84 | 0.08 | 0.04 |
| Cumulative proportion of variance explained | 0.89 | 0.98 | 0.99 | 0.84 | 0.92 | 0.96 |
| Gene of Interest | PC1 | PC2 | PC3 | PC1 | PC2 | PC3 |
| *Hsp70* | 0.06 | **0.79** | 0.41 | 0.11 | < 0.01 | 0.03 |
| *Hsc70* | **0.93** | -0.27 | 0.19 | **0.93** | -0.28 | 0.16 |
| *Hsp90* | 0.34 | 0.51 | **-0.70** | 0.29 | **0.88** | -0.06 |
| *Hsp40* | 0.14 | 0.20 | 0.21 | 0.13 | 0.05 | < 0.01 |
| *Hsp27* | 0.02 | 0.03 | 0.05 | 0.13 | 0.11 | **-0.80** |
| *GPx* | 0.01 | -0.05 | -0.34 | -0.02 | 0.22 | 0.44 |
| *CAT* | -0.02 | -0.05 | -0.37 | -0.05 | 0.27 | 0.36 |
| *SOD* | < -0.01 | < -0.01 | < -0.01 | < -0.01 | < 0.01 | < 0.01 |
| *GST* | < -0.01 | < -0.01 | -0.07 | -0.02 | 0.07 | -0.03 |
| *Nox4* | < -0.01 | < -0.01 | -0.01 | < -0.01 | 0.01 | < 0.01 |
|  | PC1 *Hsps* | PC2 *Hsps* | PC3 *Hsps* | PC1 *Hsps* | PC2 *Hsps* | PC3 *Hsps* |
| Proportion of variance explained | 0.90 | 0.08 | 0.01 | 0.87 | 0.07 | 0.03 |
| Cumulative proportion of variance explained | 0.90 | 0.98 | 0.99 | 0.87 | 0.94 | 0.97 |
| Gene of Interest | PC1 *Hsps* | PC2 *Hsps* | PC3 *Hsps* | PC1 *Hsps* | PC2 *Hsps* | PC3 *Hsps* |
| *Hsp70* | 0.06 | **0.79** | 0.52 | 0.11 | < 0.01 | 0.01 |
| *Hsc70* | **0.93** | -0.28 | 0.22 | **0.93** | -0.33 | -0.07 |
| *Hsp90* | 0.34 | 0.51 | -0.78 | 0.29 | **0.92** | -0.22 |
| *Hsp40* | 0.14 | 0.20 | 0.24 | 0.13 | 0.05 | -0.03 |
| *Hsp27* | 0.02 | 0.03 | -0.08 | 0.13 | 0.19 | **0.97** |
|  | PC1 *REs* | PC2 *REs* | PC3 *REs* | PC1 *REs* | PC2 *REs* | PC3 *REs* |
| Proportion of variance explained | 0.79 | 0.18 | 0.01 | 0.79 | 0.19 | 0.02 |
| Cumulative proportion of variance explained | 0.79 | 0.98 | 0.99 | 0.79 | 0.98 | 0.99 |
| Gene of Interest | PC1 *REs* | PC2 *REs* | PC3 *REs* | PC1 *REs* | PC2 *REs* | PC3 *REs* |
| *GPx* | **0.75** | **0.65** | 0.14 | **0.71** | **0.69** | 0.14 |
| *CAT* | **0.66** | **-0.70** | -0.28 | **0.70** | **-0.67** | -0.27 |
| *SOD* | 0.02 | -0.02 | < -0.01 | 0.02 | -0.02 | < -0.01 |
| *GST* | 0.08 | -0.30 | **0.95** | 0.09 | -0.29 | **0.95** |
| *Nox4* | 0.03 | -0.04 | -0.02 | 0.03 | -0.03 | < -0.01 |

|  | All females | | | Outliers removed | | |
| --- | --- | --- | --- | --- | --- | --- |
|  | PC1 | PC2 | PC3 | PC1 | PC2 | PC3 |
| Proportion of variance explained | 0.67 | 0.24 | 0.07 | 0.70 | 0.15 | 0.08 |
| Cumulative proportion of variance explained | 0.67 | 0.91 | 0.98 | 0.70 | 0.85 | 0.93 |
| Gene of Interest | PC1 | PC2 | PC3 | PC1 | PC2 | PC3 |
| *∆Hsp70* | 0.03 | 0.13 | **-0.95** | 0.28 | -0.41 | 0.23 |
| *∆Hsc70* | **0.95** | -0.29 | 0.01 | **0.77** | -0.14 | **-0.48** |
| *∆Hsp90* | 0.27 | **0.93** | 0.17 | 0.55 | 0.39 | **0.48** |
| *∆Hsp40* | 0.16 | 0.15 | -0.22 | 0.13 | -0.07 | 0.11 |
| *∆Hsp27* | < 0.01 | 0.13 | < -0.01 | 0.02 | **-0.61** | **0.49** |
| *∆GPx* | < 0.01 | -0.02 | 0.04 | 0.04 | 0.18 | **0.46** |
| *∆CAT* | 0.01 | 0.02 | 0.12 | 0.05 | 0.48 | 0.09 |
| *∆SOD* | < 0.01 | < 0.01 | < 0.01 | < -0.01 | < 0.01 | 0.01 |
| *∆GST* | < 0.01 | < 0.01 | 0.02 | < -0.01 | 0.12 | 0.11 |
| *∆Nox4* | < 0.01 | < 0.01 | < 0.01 | < 0.01 | 0.02 | 0.03 |
|  | PC1 *∆Hsps* | PC2 *∆Hsps* | PC3 *∆Hsps* | PC1 *∆Hsps* | PC2 *∆Hsps* | PC3 *∆Hsps* |
| Proportion of variance explained | 0.68 | 0.24 | 0.07 | 0.76 | 0.13 | 0.07 |
| Cumulative proportion of variance explained | 0.68 | 0.92 | 0.99 | 0.76 | 0.89 | 0.96 |
| Gene of Interest | PC1 *∆Hsps* | PC2 *∆Hsps* | PC3 *∆Hsps* | PC1 *∆Hsps* | PC2 *∆Hsps* | PC3 *∆Hsps* |
| *∆Hsp70* | 0.03 | 0.13 | **0.96** | 0.28 | 0.52 | < 0.01 |
| *∆Hsc70* | **0.95** | -0.29 | -0.02 | **0.78** | 0.05 | 0.54 |
| *∆Hsp90* | 0.27 | **0.93** | -0.18 | 0.54 | -0.39 | **-0.73** |
| *∆Hsp40* | 0.16 | 0.15 | 0.22 | 0.13 | 0.08 | -0.09 |
| *∆Hsp27* | < 0.01 | 0.13 | < -0.01 | 0.02 | **0.75** | -0.41 |
|  | PC1 *∆REs* | PC2 *∆REs* | PC3 *∆REs* | PC1 *∆REs* | PC2 *∆REs* | PC3 *∆REs* |
| Proportion of variance explained | 0.75 | 0.21 | 0.03 | 0.75 | 0.21 | 0.03 |
| Cumulative proportion of variance explained | 0.75 | 0.96 | 0.99 | 0.75 | 0.96 | 0.99 |
| Gene of Interest | PC1 *∆REs* | PC2 *∆REs* | PC3 *∆REs* | PC1 *∆REs* | PC2 *∆REs* | PC3 *∆REs* |
| *∆GPx* | 0.56 | **0.82** | -0.06 | 0.55 | **0.83** | -0.08 |
| *∆CAT* | **0.80** | -0.56 | -0.20 | **0.81** | -0.55 | -0.21 |
| *∆SOD* | 0.02 | < -0.01 | 0.02 | 0.02 | < 0.01 | 0.03 |
| *∆GST* | 0.19 | -0.06 | **0.97** | 0.22 | -0.05 | **0.97** |
| *∆Nox4* | 0.04 | < 0.01 | 0.09 | 0.04 | 0.02 | 0.10 |

**Supp Mat Table 3** Proportion of variance explained by and loadings of PCs 1-3 of absolute change in GOI (from early to late lactation; ∆GOI) combined; absolute change in *Hsps* and *REs* considered separately. PCs are displayed for analyses including all females (*n* = 17) and when the outliers for extreme *Hsp* values (5B, 6L and 0H) were removed (*n* = 14).

|  | PC1 *Hsps* | PC2 *Hsps* | PC3 *Hsps* |
| --- | --- | --- | --- |
| Proportion of variance explained | 0.71 | 0.16 | 0.09 |
| Cumulative proportion of variance explained | 0.71 | 0.87 | 0.96 |
| Gene of Interest | PC1 *Hsps* | PC2 *Hsps* | PC3 *Hsps* |
| log_10_ *Hsp70* | 0.49 | **0.85** | 0.18 |
| log_10_ *Hsc70* | 0.40 | -0.09 | -0.51 |
| log_10_ *Hsp90* | **0.50** | -0.35 | -0.02 |
| log_10_ *Hsp40* | 0.32 | -0.05 | **-0.57** |
| log_10_ *Hsp27* | **0.50** | -0.38 | **0.62** |
|  | PC1 *REs* | PC2 *REs* | PC3 *REs* |
| Proportion of variance explained | 0.85 | 0.08 | 0.04 |
| Cumulative proportion of variance explained | 0.85 | 0.93 | 0.97 |
|  | PC1 *REs* | PC2 *REs* | PC3 *REs* |
| log_10_ *GPx* | **0.81** | -0.16 | -0.47 |
| log_10_ *CAT* | 0.26 | -0.28 | **0.83** |
| log_10_ *SOD* | 0.40 | -0.29 | 0.18 |
| log_10_ *GST* | 0.35 | **0.90** | 0.24 |
| log_10_ *Nox4* | < 0.01 | < 0.01 | < 0.01 |

**Supp Mat Table 4** Proportion of variance explained by and loadings of PCs 1-3 lactating-fasting mothers and foraging (presumed pregnant) females combined; *Hsps* and *REs* considered separately. PCs are displayed for analyses including all females (*n* = 17 [lactating-fasting]; *n* = 13 [foraging (presumed pregnant)]). Relative mRNA abundance data were log10 transformed prior to the analyses.
